# Cofilin-induced cooperative conformational changes of actin subunits revealed using cofilin-actin fusion protein

**DOI:** 10.1038/srep20406

**Published:** 2016-02-04

**Authors:** Nobuhisa Umeki, Keiko Hirose, Taro Q. P. Uyeda

**Affiliations:** 1 Biomedical Research Institute, National Institute of Advanced Industrial Science and Technology, Tsukuba, Ibaraki 305-8562, Japan; 2 Graduate School of Life and Environmental Sciences, University of Tsukuba, Ibaraki 305-8572, Japan; 3 Cellular Informatics Lab., RIKEN, Wako, Saitama 351-0198, Japan

## Abstract

To investigate cooperative conformational changes of actin filaments induced by cofilin binding, we engineered a fusion protein made of *Dictyostelium* cofilin and actin. The filaments of the fusion protein were functionally similar to actin filaments bound with cofilin in that they did not bind rhodamine-phalloidin, had quenched fluorescence of pyrene attached to Cys374 and showed enhanced susceptibility of the DNase loop to cleavage by subtilisin. Quantitative analyses of copolymers made of different ratios of the fusion protein and control actin further demonstrated that the fusion protein affects the structure of multiple neighboring actin subunits in copolymers. Based on these and other recent related studies, we propose a mechanism by which conformational changes induced by cofilin binding is propagated unidirectionally to the pointed ends of the filaments, and cofilin clusters grow unidirectionally to the pointed ends following this path. Interestingly, the fusion protein was unable to copolymerize with control actin at pH 6.5 and low ionic strength, suggesting that the structural difference between the actin moiety in the fusion protein and control actin is pH-sensitive.

Actin filaments play critical functions in a variety of cellular activities such as cytokinesis, lamellipodial extension, adhesion, intracellular transport and nuclear functions^1^. Those diverse functions depend on interactions with specific actin binding proteins (ABPs) at specific sites within a cell. A major question that remains in cell biology is how the activities of each ABP are spatially and temporally regulated such that actin filaments can perform different multiple functions simultaneously in a cell. In a number of cases, biochemical signaling, such as phosphorylation and changes in the concentration of signaling molecules, have been implicated in local and specific regulation of ABPs (e.g.,^2^,^3^,^4^,^5^). However, not all the localized activities of ABPs are fully explained by such specific biochemical signaling.

Separately, biochemical and biophysical evidence has accumulated to show that binding of ABPs induces specific conformational changes in actin filaments. In certain cases, ABP-induced conformational changes that occurred in the bound actin subunit were shown to propagate to neighboring subunits in the same filament. Those cooperative conformation changes, a special case of allosteric conformational changes involving multiple neighboring subunits in filaments, may have functional implications since the affected actin subunits would have altered affinities for various ABPs. Furthermore, if the affected actin subunits have higher affinity for a specific ABP that initially bound to the filament, it would result in cooperative binding of that ABP along the filament. This domino effect has been demonstrated for cofilin binding to actin filaments in which cofilin binds to actin filaments cooperatively, and forms clusters along the filament while leaving other parts of the filament bare^6^,^7^,^8^. The helical pitch of the actin filaments in the cofilin clusters is 25% shorter than the control filaments^6^,^7^, and image analysis suggested that the neighboring bare zone is as supertwisted as in the clusters^6^. Recently, high-speed atomic force microscopy (HS-AFM) demonstrated that approximately a half helical pitch, containing 14 actin subunits, of the bare zone on the pointed end side of a cofilin cluster is supertwisted, and the cofilin cluster grows into this supertwisted bare zone^9^. Unidirectional cooperative conformational changes in actin filaments induced by cofilin clusters are supported by a more recent observation by Gressin *et al*. that severing of filaments occurs only on the pointed-end side of cofilin clusters^10^. Cooperative conformational changes of actin subunits induced by cofilin binding have also been detected by more indirect biophysical assays, such as differential scanning calorimetry and time-resolved phosphorescence anisotropy, and those studies found that neighboring actin subunits in the order of 10^2^ are affected by each bound cofilin molecule^11^,^12^,^13^. The number of affected neighboring actin subunits differs significantly between our AFM measurements and previous biophysical estimates, suggesting that there are at least two distinct types of cofilin-induced cooperative conformational changes of actin subunits. Obviously, further studies are needed to elucidate the consequences that binding of this essential ABP has on neighboring actin subunits.

Myosin is another major family of ABPs with a long research history. Those studies demonstrated that binding of the motor domain of myosin II also evokes cooperative conformational changes to neighboring actin subunits^14^,^15^,^16^,^17^. Furthermore, under certain conditions, myosin binding to actin filaments is cooperative^18^,^19^, suggesting the possibility that myosin also uses cooperative conformational changes of neighboring actin subunits for its cooperative binding. To explore this possibility, we previously developed a fusion protein of actin and the motor domain of myosin II (S1)^20^. Biochemical and electron microscopic characterization revealed that the actin-S1 fusion protein copolymerizes with control actin and acts as a covalently crosslinked mimic of the natural complex of actin and S1^20^. Furthermore, the fusion protein proved very useful in examining S1-induced cooperative conformational changes of actin filaments since the fusion protein approach enabled straightforward estimation of the ratio of S1-bound and free actin subunits in copolymers^21^. Use of this fusion protein has an additional advantage that one can expect to obtain relatively homogeneous mixture of S1-bound and free actin subunits in copolymers, while S1 binds cooperatively to actin filaments when it is added to solutions of actin filaments.

Here, to take those advantages of the fusion protein approach in investigating cooperative conformational changes of actin filaments induced by cofilin binding, we developed and characterized a fusion protein between cofilin and actin. The results demonstrate that the cofilin-actin fusion protein behaves as a mimic of a covalently crosslinked actin-cofilin complex, and affects the structure of neighboring actin subunits within copolymers, similar to actin-S1 fusion protein. We propose that the cofilin-actin fusion protein is a useful tool to study the mechanism and functional significance of cofilin-induced cooperative conformational changes of actin filaments.

## Results

### Polymerization of cofilin-actin fusion protein

**Figure 1:**
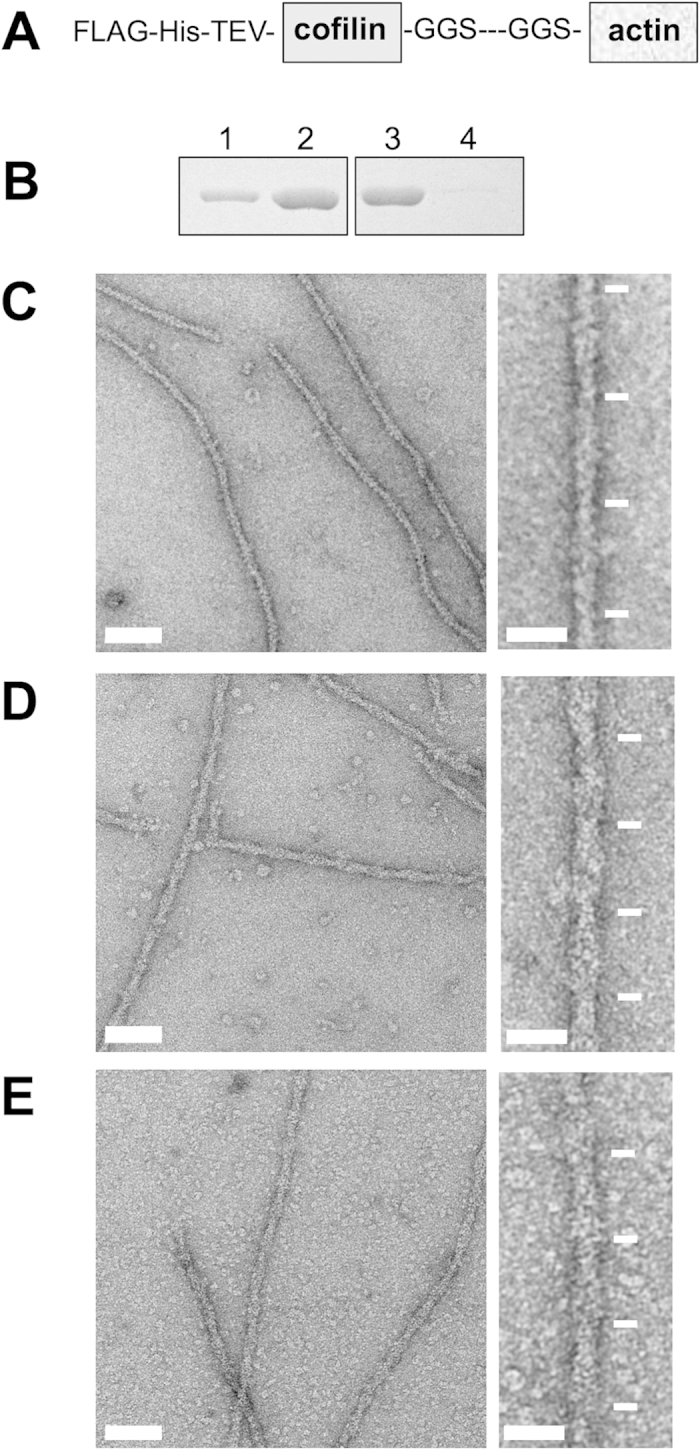
Polymerization of the cofilin-actin fusion protein. (**A**) Schematic structure of the cofilin-actin fusion protein. (**B**) Reversible polymerization and depolymerization of the fusion protein. Lane 1 and 2 are sup and pellet fractions after ultracentrifugation of 5 μM cofilin-actin fusion protein in buffer (100 mM KCl, 20 mM Pipes pH 6.5, 2.5 mM MgCl_2_, 0.5 mM EGTA, 0.5 mM ATP and 1 mM DTT), respectively. The pellet fraction was dissolved and dialyzed against G buffer, and ultracentrifuged again. Lane 3 and 4 are sup and pellet fractions of the second ultracentrifugation, respectively. (**C–E**) Electron micrographs of negatively stained filaments of control actin (**C**) and the fusion protein (**D**). (**E**) shows control actin filaments decorated with five-fold excess *Dictyostelium* cofilin. Bars in the right image of each pair show approximate positions of the crossover points. Scale bar: 50 nm (left images), 20 nm (right images).

**Figure 2:**
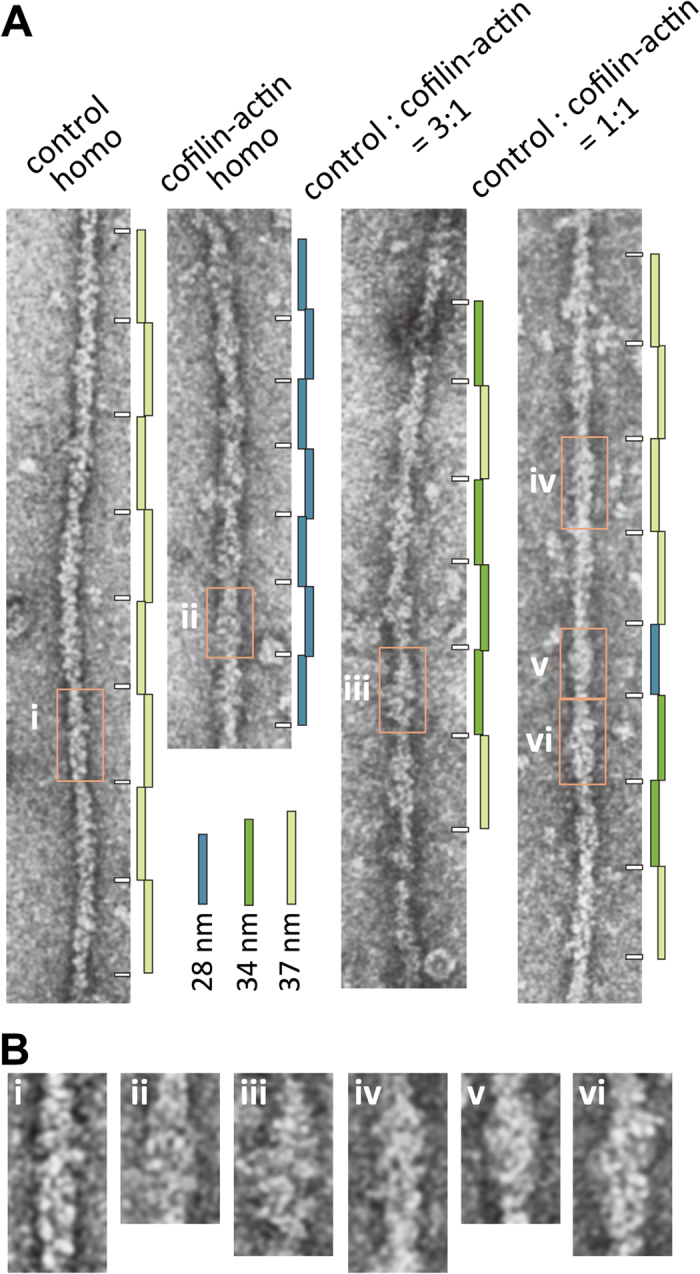
Electron micrographs of negatively stained copolymers. (**B**) shows the enlarged views of the boxed regions in (**A**). When control actin and cofilin-actin fusion protein were mixed and then allowed to polymerize, the thickness and the half helical pitch of the resulting filaments were variable along each filament. Some segments (e.g., segment iv) were similar to the control actin filament (i), while other segments (v) along the same filament looked like the cofilin-actin filament (ii). However, there was no discernible boundary between the control-actin-like segments and cofilin-actin-like segments. Some sections of the mixed filaments (e.g., (iii) and (vi)) had an intermediate half helical pitch, for example, ~34 nm. The surface of the filament in these regions looked rough, suggesting sparsely distributed cofilin-actin (i.e., copolymerization). When a half helical pitch was shorter, the neighboring 1 or 2 half helical repeats tended to have a shorter pitch, but the effect did not propagate for a long distance.

Figure 1A shows the organization of the cofilin-actin fusion protein, in which *Dictyostelium* actin was fused to the C-terminus of *Dictyostelium* cofilin via a Gly-based 14 amino acid residue linker. This cofilin-actin fusion protein was expressed in *Dictyostelium* cells, and purified using the N-terminal FLAG and His tags. Ultracentrifugation demonstrated that cofilin-actin polymerized and depolymerized normally in a salt-dependent manner (Fig. 1B). This is consistent with the previous finding that saturating concentrations of cofilin promotes, rather than inhibits, polymerization of actin by accelerating the nucleation process^22^. In electron micrographs of negatively stained samples, the filaments of cofilin-actin appeared thicker, and the half helical pitch was shorter (~28 nm), than the control actin filaments (Fig. 1C,D). This is consistent with earlier observations of actin filaments fully bound with cofilin^6^,^7^,^9^ (Fig. 1E). When the control actin and cofilin-actin fusion protein were mixed in G buffer and then allowed to copolymerize, the thickness and the half helical pitch of the resulting filaments were variable along each filament (Fig. 2). There were sections with half helical pitch of ~37 nm and ~28 nm, which are the half helical pitches of control actin filaments and those fully decorated with cofilin, respectively^6^,^7^, and some other sections had intermediate half helical pitch around 34 nm. These results suggest that control actin and cofilin-actin fusion protein do not homogeneously mix in copolymers. Sections of 28 nm half helical pitch were thicker than control actin filaments, and the surface of the sections with intermediate half helical pitch looked rough, suggesting the presence of sparsely-distributed cofilin moieties of the fusion protein. However, we were unable to identify boundaries between sections of different half helical pitches, because the spatial resolution of half helical pitch measurement cannot be shorter than half helical pitches, and also because individual cofilin moieties in cofilin-actin fusion protein were too small to identify unambiguously. When the half helical pitch was shorter, the neighboring 1 or 2 repeats tended to have a shorter pitch, but the effect did not propagate for a long distance.

Fluorescence microscopy of Alexa-labeled proteins also showed that cofilin-actin forms filaments, although the filaments of cofilin-actin were significantly shorter than those of control actin (Supplementary Figs S1A and S2). We speculate that this was due to the nucleation-promoting activity of the cofilin moiety in the fusion protein. Indeed, cofilin-actin fusion protein formed filaments extremely rapidly, as measured by the increase in light scattering (Supplementary Fig. S3). This finding is consistent with previous studies that cofilin/actin complexes form filaments more rapidly compared with actin alone^22^,^23^,^24^.

Fluorescence microscopy further demonstrated copolymerization of cofilin-actin with control actin when the two proteins were mixed in G buffer and induced to polymerize at 100 mM KCl and pH 7.4 (Supplementary Figs S1B and C). Intriguingly, the two proteins hardly copolymerized when mixed in G-buffer and induced to polymerize at 25 mM KCl and at a lower pH of 6.5 (Supplementary Fig. S1D). This means that, at 25 mM KCl and pH 6.5, a condition known to increase the affinity between actin filaments and cofilin^25^,^26^,^27^, monomers of cofilin-actin bind to the ends of polymers made of cofilin-actin, but not to the ends of filaments made of control actin. Conversely, monomeric control actin binds to the ends of polymers made of control actin, but not to the ends of filaments made of cofilin-actin. Related to this, Andrianantoandro and Pollard^22^ suggested that saturating concentrations of cofilin added to a solution of monomeric actin stabilizes “long-pitch dimer” of actin. If actin molecules in those dimers have the conformation similar to those in fully cofilin-decorated actin filaments, and if cofilin-actin fusion protein tends to form similar long-pitch dimers or longer oligomers in G-buffer, they may have a higher affinity for filaments of cofilin-actin than for control actin.

### Cofilin moiety in the fusion protein has severing and depolymerizing activities

The major known effects of cofilin on actin filaments are severing and depolymerization. To assess whether the cofilin moiety in the fusion protein has those activities, we performed sedimentation assay and electron microscopic observation (Supplementary Fig. S4). As mentioned above, cofilin-actin and control actin hardly copolymerize when mixed in G-buffer at 25 mM KCl and pH of 6.5, whereas copolymerization was inducible at pH 7.4. Therefore, in order to perform sedimentation assay and electron microscopic observation using copolymers at pH 6.5, cofilin-actin and control actin were copolymerized at pH 7.4. Then concentrated Pipes buffer was added to lower the pH to 6.5. After the copolymerization, depolymerization was induced by the addition of concentrated Hepes buffer pH 8.3, and the polymeric and depolymerized fractions were separated by ultracentrifugation followed by SDS-PAGE (Supplementary Fig. S4A). The amount of cofilin-actin in the supernatant fraction was significantly higher at pH 8.3 than at pH 6.5. This result suggested that cofilin moiety in the fusion protein has a pH-sensitive depolymerization activity, as does cofilin^28^. We also checked severing events of copolymers by electron microscopy. Some copolymer filaments showed discontinuities (Supplementary Fig. S4B), which appeared to be caused by severing after the filaments were loosely immobilized on electron microscopy grids.

### Cofilin-actin fusion protein induces cooperative conformational changes in neighboring actin subunits

**Figure 3:**
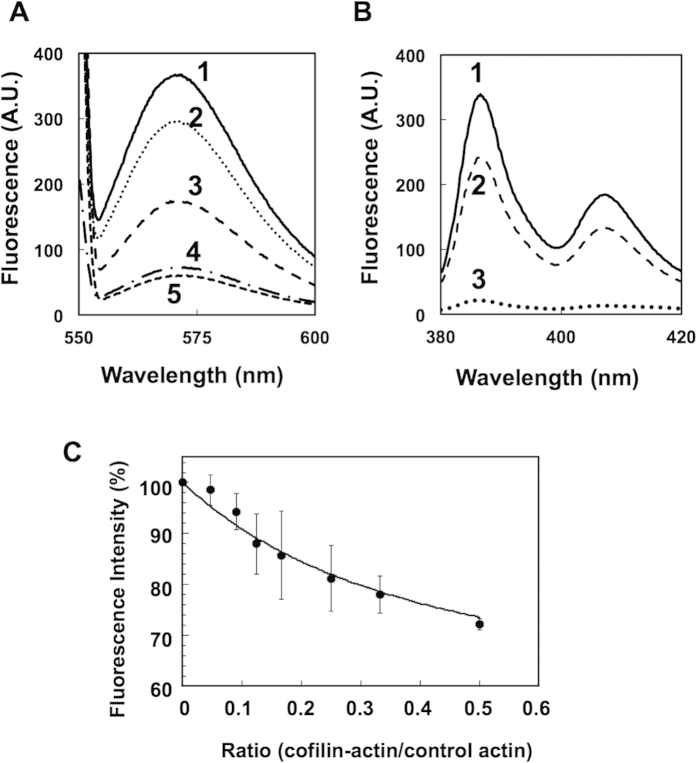
(**A**) Fluorescence emission spectrum of mixtures of rhodamine-phalloidin and filaments. Rhodamine-phalloidin at 1 μM was added to 4 μM control actin filaments (line #1), copolymer of 4 μM control actin and 4 μM cofilin-actin (line #2), 4 μM control actin and 10 μM cofilin (line #3), and 4 μM cofilin-actin filaments (line #4). The bottom (line #5) is a control of 1 μM rhodamine-phalloidin only in buffer. (**B,C**) Fluorescence of pyrene attached to actin. (**B**) The upper solid line (line #1) shows the emission spectrum of 3 μM pyrene-labeled control actin filaments, and the middle line (line #2) is that of copolymers of 3 μM pyrene-labeled control actin and 3 μM cofilin-actin fusion protein. The lower line (line #3) shows the emission spectrum of 3 μM pyrene-labeled monomeric control actin in G-buffer. This result is consistent with previous report that the fluorescence intensity of pyrene-F-actin decreased with cofilin binding^39^. (**C**) shows the fluorescence intensities (emission at 407 nm) of copolymers of 3 μM pyrene-control actin and various concentrations of the fusion protein (mean ± SD, N = 3). The line is the best fit curve using the equation described in the main text.

That the cofilin moiety in cofilin-actin is functional was further confirmed using rhodamine-phalloidin (rh-ph). Consistent with a previous report^29^, the addition of 1 μM rh-ph to 4 μM control actin filaments significantly increased the fluorescence intensity when compared with buffer (Fig. 3A, #1 and #5). In contrast, the addition of rh-ph to filaments of 4 μM fusion protein hardly increased the fluorescence intensity (Fig. 3A, #4). Preincubation of 4 μM control actin filaments with 10 μM cofilin strongly inhibited the increase in fluorescence when rh-ph was added subsequently, but not to the level of cofilin-actin filaments. These results are consistent with the notion that the cofilin moiety in cofilin-actin efficiently and properly interacted with the actin moiety, and inhibited binding of rh-ph, just as cofilin does^29^. Notably, when 4 μM control actin was copolymerized with 4 μM cofilin-actin, the increase in fluorescence of rh-ph was lower than that in 4 μM actin alone (Fig. 3A, #2). This indicates that one mole of the cofilin moiety of cofilin-actin rendered more than one filament subunit, either the control actin or the actin moiety of cofilin-actin, inaccessible to rh-ph.

Fluorescence assays using pyrene-labeled actin also demonstrated allosteric conformational changes evoked by the cofilin-actin fusion protein. Figure 3B shows that the fluorescence intensity of the copolymer (3 μM pyrene-labeled control actin and 3 μM unlabeled cofilin-actin fusion protein) was significantly lower than that of 3 μM pyrene-labeled control actin filaments. To estimate how many pyrene-labeled control actin subunits were affected by each molecule of cofilin-actin in copolymers, we examined the extent to which pyrene fluorescence was quenched when a fixed concentration of pyrene-labeled control actin was copolymerized with different concentrations of cofilin-actin (Fig. 3C). To perform quantitative fitting of the result, two simple assumptions were made: (i) the pyrene-labeled control actin and cofilin-actin are homogenously mixed in the copolymers; (ii) each fusion protein molecule quenches the pyrene fluorescence of a control actin subunit by a factor of X if the control actin is within the Nth neighbor on either side, but does not affect actin subunits beyond the Nth neighbor. In other words, each cofilin-actin molecule is assumed to affect 2N neighbor control subunits. Based on these assumptions, and using the method of Visegrady *et al*.^30^, the overall fluorescence intensity of copolymers is given by:


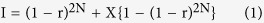


where I is the normalized fluorescence intensity, r is the molar fraction of cofilin-actin to the total actin subunits, i.e., the sum of cofilin-actin and control actin. Fitting of the data with the equation yielded 2N = 2.7 and X = 0.60 (Fig. 3C). This value of N is potentially an underestimate, if the fusion protein is not homogeneously distributed in the copolymers. Nonetheless, this is larger than 1, and therefore, we concluded that cofilin-actin evokes cooperative conformational changes to multiple neighboring actin subunits in copolymers. For the sake of simplicity, we assumed a bidirectional propagation of the conformational change, but the same result is reached if unidirectional propagation is assumed (data not shown).

### *S*ubtilisin cleavage assay

**Figure 4:**
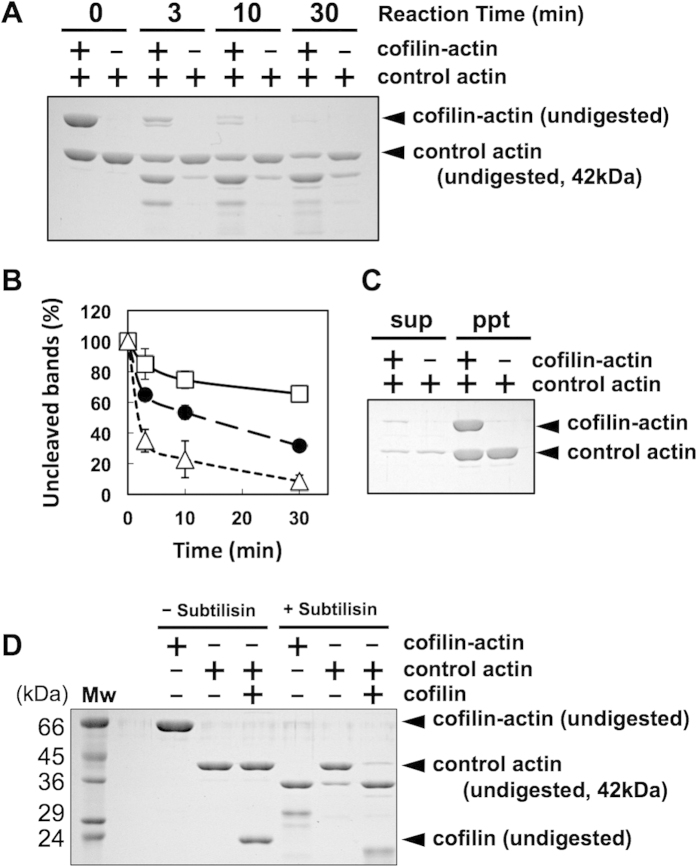
Rates of cleavage by subtilisin. (**A**) Copolymer of 5 μM control actin and 5 μM cofilin-actin fusion protein or homopolymer of 5 μM control actin was digested with 5 μg/mL subtilisin for the indicated time, and analyzed by SDS-PAGE. (**B**) is the quantitation (mean ± SD, N = 3) of intact actin in control actin homopolymer (open squares), intact actin in copolymer (filled circles) and intact cofilin-actin in copolymer (open triangles). (**C**) shows the sup and pellet fractions after ultracentrifugation of the copolymer and homopolymer filaments, respectively, before treatment with subtilisin, showing that polymerization was almost complete in both samples. (**D**) Digestion of the cofilin-actin fusion protein homopolymer. Digestion of cofilin-actin did not yield a 42 kDa band, demonstrating that the 42 kDa band seen in (**A**) and Fig. 5A are all intact actin, rather than a digestion product of cofilin-actin.

Subtilisin slowly cleaves the DNase loop of actin in filaments, but when cofilin is bound, the cleavage reaction is significantly accelerated^31^. We thus employed a subtilisin-cleavage assay to further examine if conformational changes of control actin subunits induced by neighboring cofilin-actin fusion protein are cooperative. Under our experimental conditions, 65.6% ± 5.1% (mean ± SD, N = 3) of the control actin subunits in homopolymers remained intact after 30 min of the reaction, while cofilin-actin in the copolymers was almost completely digested under the same condition (Fig. 4A,B). The control actin subunits in copolymers showed an intermediate level of sensitivity to subtilisin cleavage (31.7% ± 1.1% remained intact), demonstrating that cofilin-actin affected the conformation of neighboring control actin subunits in copolymers. Figure 4C shows that in both samples control actin and cofilin-actin were normally polymerized.

**Figure 5:**
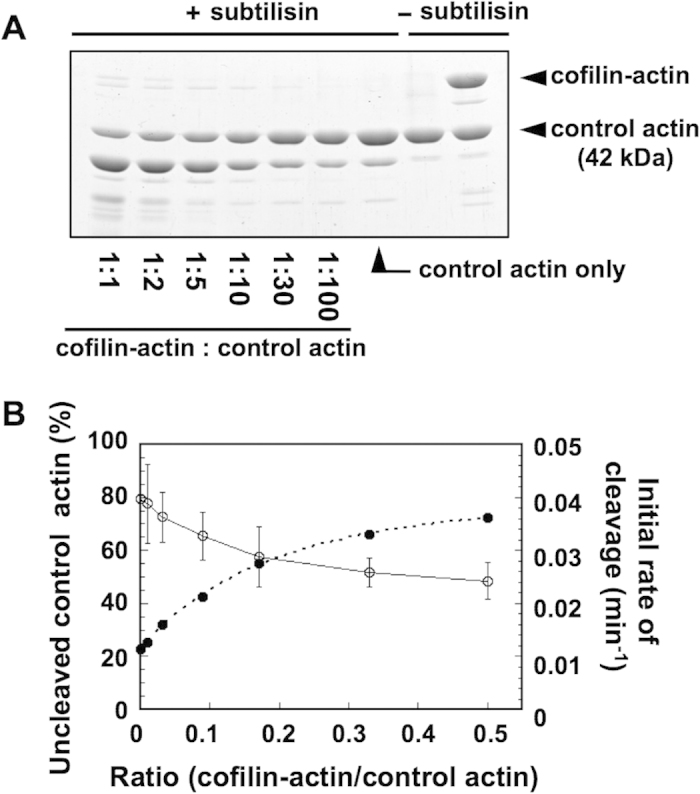
Dependence of DNase loop cleavage rate on the molar ratio of cofilin-actin to control actin. (**A**) Copolymers of 5 μM control actin and various concentrations of cofilin-actin were digested with 8.3 μg/mL subtilisin for 25 min, and analyzed by SDS-PAGE. (**B**) is the quantitation (mean ± SD, N = 3), and the calculated initial rate of cleavage. The broken line shows the best fit curve using the equation described in the main text.

To estimate how many control actin subunits were affected by each molecule of cofilin-actin in copolymers, we prepared copolymers with different molar ratios of cofilin-actin to control actin, and measured the rate of cleavage of control actin subunits. Figure 5A shows a general trend in which the cleavage rate became faster as the molar ratio became larger. The fraction of uncleaved control actin was plotted against the molar ratio in Fig. 5B. From this data, we estimated the initial rate of cleavage *k* using the following equation:


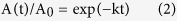


where A(t) is the concentration of uncleaved control actin at t and A_0_ is the initial concentration of control actin. Again, if the fusion protein was randomly distributed within the copolymer, and each molecule of the fusion protein affected the conformation of N neighboring control actin subunits on either side of it, and the affected control actin subunits are cleaved at a rate of *k*_1_ whereas the unaffected subunits are cleaved at a rate of *k*_0_, then the overall cleavage rate *k* is given by:


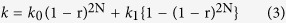


where r is the molar fraction of the fusion protein in total actin subunits. Fitting of the relationship between *k* and r using this equation yielded 2N = 5.3 (Fig. 5B). As in the case of quenching of pyrene fluorescence, this value of 2N is potentially an underestimate. Furthermore, if propagation of the conformational changes induced by cofilin-actin is perturbed beyond the cleaved subunit, this would also apparently shorten the range of affected segment of the copolymer filaments. Nonetheless, again, the value of 2N = 5.3 is much larger than 1, and therefore, we concluded that cofilin-actin evokes cooperative conformational changes to multiple neighboring actin subunits in copolymers.

**Figure 6:**
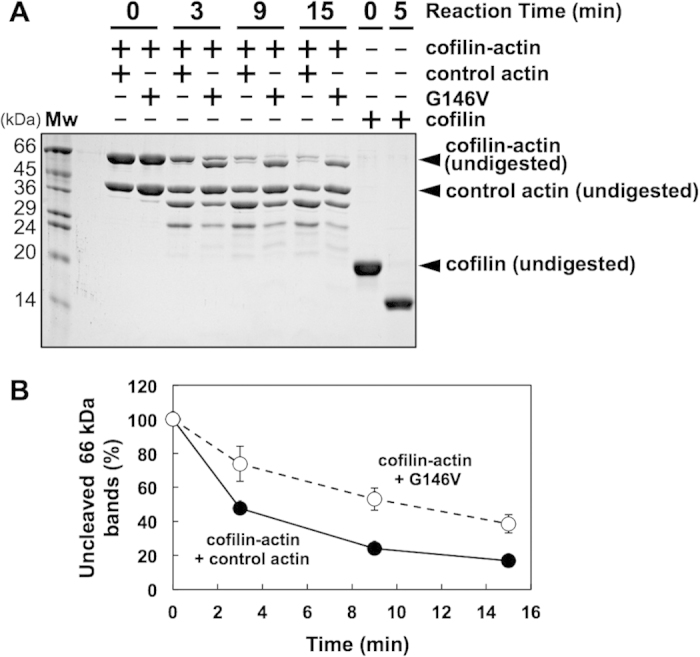
Effects of copolymerization with G146V mutant actin on the cleavage of DNase loop in the cofilin-actin fusion protein. Cofilin-actin with intact DNase loop became slightly smaller after treatment with subtilisin, presumably due to nicking in the cofilin moiety, as cofilin was readily nicked by subtilisin (compare the two lanes at the right end of Fig. 6A). (**B**) Quantitation of the 66 kDa bands includes both intact and nicked bands.

We previously showed that a G146V mutant actin is unable to bind cofilin, and copolymerization of G146V actin with wild type actin renders the wild type actin inaccessible to cofilin, even at a G146V:wild type = 1:10 molar ratio^32^. This suggests that cofilin binding depends on long-range cooperative conformational changes of the filament, which is inhibited by scattered G146V subunits. Alternatively, the G146V subunits affected the structure of more than 10 neighboring wild type subunits and reduced their affinity for cofilin. In either case, long-range cooperative conformational changes are involved in this G146V mutant actin-mediated inhibition of cofilin binding. We thus queried whether copolymerization of cofilin-actin with G146V actin protects its DNase loop from subtilisin cleavage (Fig. 6). Under the present condition, cofilin-actin in a copolymer with control actin (wild-type) was almost completely digested. In contrast, a significant fraction of cofilin-actin retained an intact DNase loop when copolymerized with G146V mutant actin. This result demonstrates that the cooperative conformational changes involving cofilin-actin are bi-directional, in that not only cofilin-actin affects the conformation of the neighboring actin subunits in a copolymer, but also the neighboring actin subunits affect the conformation of the actin moiety in cofilin-actin.

### Cosedimentation assay

**Figure 7:**
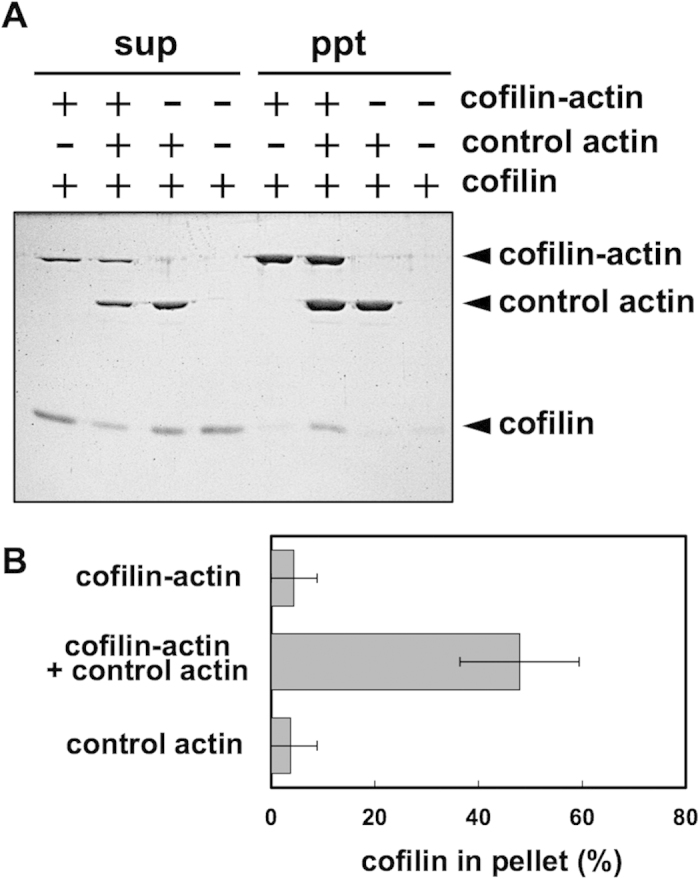
Cosedimentation assays to demonstrate cooperative binding of cofilin. First, 3 μM cofilin-actin fusion protein and 1.5 μM control actin were mixed in G-buffer as indicated, and then allowed to polymerize in buffer (160 mM KCl, 2 mM Hepes pH 7.4, 2 mM MgCl_2_, 0.4 mM EGTA, and 0.2 mM ATP). Subsequently, 2 μM cofilin and 20 mM Pipes pH 6.5 was added as indicated and ultracentrifuged, and the resultant sup and pellet fractions were subjected to SDS-PAGE. (**B**) shows the quantitation (mean ± SD, N = 3).

Finally, we attempted to recapitulate the cooperative binding of cofilin to actin filaments^6^,^7^,^8^ in cosedimentation assays using cofilin-actin. At pH 6.5 and a KCl concentration of 160 mM, 2 μM of cofilin hardly cosedimented with 3 μM of control actin filaments (Fig. 7). As expected, when 1.5 μM of cofilin-actin was copolymerized with 3 μM of control actin, the same concentration of cofilin efficiently cosedimented with the copolymer filaments (Fig. 7 and Supplementary Fig. S5). Since bound cofilin molecules do not directly touch each other along actin filaments^33^, this enhanced binding of cofilin to the copolymers must involve allosteric conformational changes of control actin subunits induced by the fusion protein.

## Discussion

We have developed a novel chimeric protein that fuses cofilin and actin, and showed that both the cofilin and actin moieties of the fusion protein function properly, in that the actin moiety polymerizes reversibly in a salt-dependent manner (Fig. 1B), the cofilin moiety has nucleation promoting activity (Supplementary Figs S1A and S3), inhibits phalloidin binding (Fig. 3A), quenches the fluorescence of pyrene (Fig. 3B) and promotes cleavage of the DNase loop by subtilisin (Fig. 4). More importantly, when copolymerized with control actin, the fusion protein affected the conformation of multiple molecules of neighboring control actin subunits (cooperative conformational changes; Figs 3 and 5).

Interpretation of two aspects of the effects of the fusion protein on neighboring actin subunits requires caution, however. First, the fusion protein and control actin do not form homogeneously mixed copolymers, as demonstrated by electron microscopy. Additionally, it is evident from fluorescence microscopic observations that the fusion protein and control actin do not copolymerize and form separate filaments at pH 6.5 and 25 mM KCl. We suspect that this is because the structural difference between the actin moiety in cofilin-actin and control actin is pH-dependent, being too large to copolymerize at pH 6.5, but not so at pH 7.4. In this scenario, the two proteins become incorporated into copolymers at pH 7.4, but do not mix homogeneously due to subtle structural differences. If the two proteins do not mix homogeneously in copolymers, that would complicate the quantitative interpretation of the consequences of incorporating cofilin-actin into copolymers. The number of affected control actin subunits may be much larger than estimated assuming homogenous mixing. More quantitative data interpretation would be possible if we can find a polymerization condition under which the cofilin-actin fusion protein and control actin are mixed homogenously in copolymers.

**Figure 8:**
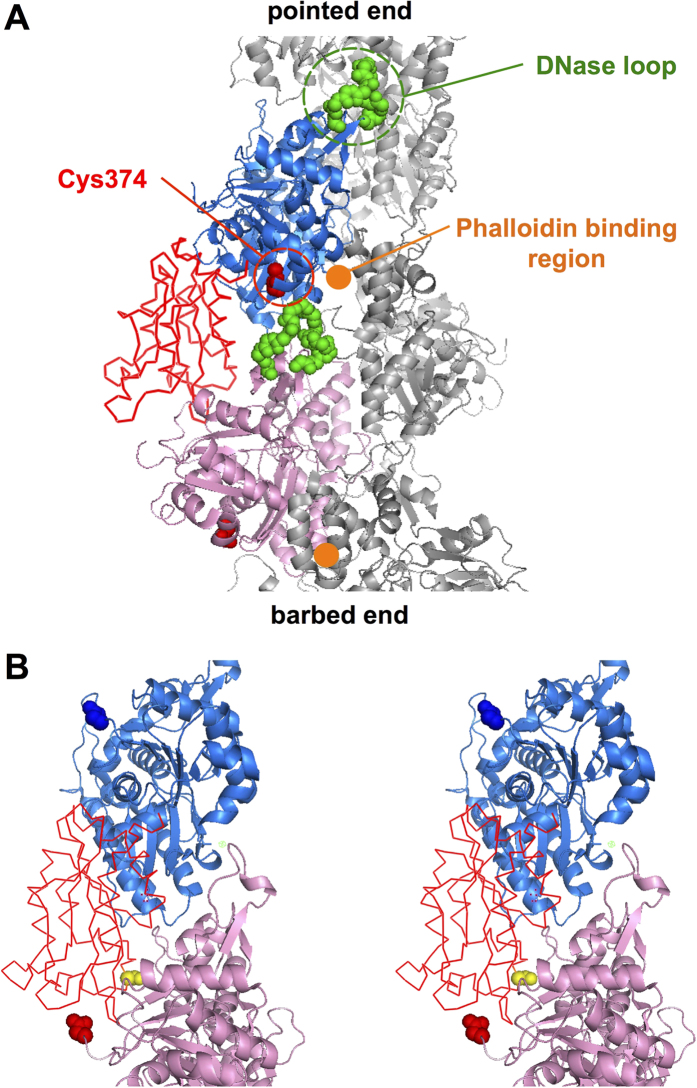
Positional relationship between the actin and cofilin moieties of the cofilin-actin fusion protein and a control actin subunit in a copolymer. (**A**) Within the structure of the actin-cofilin complex obtained by high-resolution electron microscopic analyses (PDB ID:3J0S,^33^), two actin subunits and a cofilin molecule bridging the two are shown in blue and pink ribbon representations and a red wire-frame model, respectively. Additionally, the DNase loops and Cys374 of the two actin subunits are shown in green and red space-filling models, respectively, and the approximate phalloidin binding sites^40^,^41^ are marked by orange dots. (**B**) Stereo view of 3D structure of actin-cofilin complex. Se156 of cofilin is shown in a yellow space-filling model. The N-termini of the two actin subunits are shown in red and blue space-filling models, respectively.

Second, we need to know the spatial relationship among the actin moiety of the fusion protein, control actin and the cofilin moiety of the fusion protein, where the fusion protein and control actin abut on each other. High-resolution electron microscopy revealed that a cofilin molecule binds to two adjacent actin subunits in the longitudinal strand^7^,^33^. The question is which of the two actin subunits, either the one on the pointed end side or on the barbed end side, is the actin moiety of the fusion protein when the fusion protein abuts actin subunits in a copolymer. In the fusion protein, the C-terminus of *Dictyostelium* cofilin was fused to the N-terminus of *Dictyostelium* actin via a Gly-based 14 amino acid residue linker. The C-terminal residue of *Dictyostelium* cofilin corresponds to Ser156 of human cofilin 2 used by Galkin *et al*. in their electron microscopic study^33^, and in the structure of the complex, the distance between Ser156 of human cofilin and the N-terminus of the actin subunit on the pointed end side is 5.85 nm, while that on the barbed end side is 3.11 nm. The maximally stretched length of a 14-residue polypeptide is 5.32 nm^34^, and therefore, placing the actin moiety of the fusion protein on the barbed end side of the cofilin moiety is by far energetically favorable. We thus conjectured that in the majority of cases, the control actin subunit is placed on the pointed end side of the cofilin moiety of the fusion protein (Fig. 8). How then does this relate to the allosteric effect of the fusion protein on neighboring actin subunits?

Using HS-AFM, we recently discovered that the growth of cofilin clusters along actin filaments is unidirectional toward the pointed end of the filament^9^. Thus, when a new cofilin molecule binds to a copolymer filament near a fusion protein molecule during cooperative binding (Fig. 7), we speculate that the new cofilin molecule binds to the pointed end face of the actin subunit on the pointed end side of the cofilin moiety. The DNase loop, whose susceptibility to subtilisin is enhanced when actin is copolymerized with the fusion protein, is on the pointed-end face of the molecule. Therefore, binding of the cofilin moiety of the fusion protein to the barbed end face of the actin subunit presumably causes both separation of the tips of the two domains due to a propeller motion between the two domains and a conformational change of the DNase loop, which previous high-resolution electron microscopy observed in actin subunits fully complexed with cofilin^33^. It should be emphasized here that the resultant conformational change at the pointed-end face of the affected actin subunit is very large, while that on the barbed end face is relatively subtle. A binding site for cofilin spans over two actin subunits^33^ (Fig. 8), and in theory, the binding affinity can be modulated by conformational changes of either subunit that constitutes the binding site. However, because the magnitude of conformational changes is much larger on the pointed end face of the affected actin subunit, we can reasonably assume that the affinity is primarily modulated by conformational changes of the pointed end face of the actin subunit on the barbed end side of the binding site. This would provide a simple explanation why cofilin clusters grow only to the pointed ends.

Fitting of the dependence of the rate of DNase loop cleavage on the molar fraction of the fusion protein in copolymers suggests that each fusion protein affects the susceptibility of 5.4 neighboring control actin subunits. This fitting was performed based on the assumption that the cooperative conformational changes propagate to both directions originating from the fusion protein, but since cofilin clusters grow unidirectionally to the pointed ends, it is probably more realistic to assume that the cooperative conformational changes are propagated to the pointed ends only. It is tempting to further assume that the conformational changes that occurred in the control actin subunit on the pointed end side of the fusion protein induce the same conformational change to the next control actin subunit on the pointed end side, and this is repeated on average 4.4 times. This view is consistent with our recent HS-AFM observation^9^, which revealed that the supertwisted structure of actin filaments, a hallmark of cofilin-bound actin filaments^7^, is propagated to the neighboring bare zone on the pointed end side of a cofilin cluster. The length of the supertwisted bare zone was roughly one half helical pitch, or approximately 7 actin subunits along each strand.

In this scenario, a newly bound cofilin molecule is likely to bind to any of the two consecutive actin subunits in the 5.4 affected neighboring free actin subunits during cooperative binding. This prediction is qualitatively consistent with the recent high-resolution single molecule cofilin binding assay, which concluded that after the first fluorescent cofilin molecule is bound to an actin filament, the second fluorescent cofilin molecule is likely to bind to within 65 nm of the first molecule but not necessarily immediately next to the first molecule^35^. There are 13 actin subunits in each protofilament within the 65 nm region, which is ~two-fold more than our estimate of 5.4. This difference may be due to an underestimate of the number of affected control actin subunits due to inhomogeneous mixing of control actin and the fusion protein, and/or differences in detection methods. Clearly more work is needed to elucidate the mechanism of cooperative binding of cofilin to actin filaments and the accompanying conformational changes of the filaments, including establishing methods to prepare a homogeneously mixed copolymer of actin and the fusion protein.

## Methods

### Plasmid Construction

The fusion gene that encodes cofilin-actin was constructed in multiple steps of restriction enzyme-mediated cloning using the FLAG-His-TEV encoding sequence generated by the mutual priming method, the PCR-amplified *Dictyostelium cofA* gene and the pTIKLAR plasmid^36^, which expresses *Dictyostelium act15* gene under the control of the *act15* promoter. The resultant expression vector was named pTIKL cofilin-AR. The amino acid sequence upstream of the cofilin moiety is *MDYKDDDDKGSSHHHHHHHHGSSENLYFQGDG*MSSGI…, where the italics show a FALG tag, a His tag followed by a TEV cleavage site, and MSSGI is the N-terminal sequence of CofA. The sequence between CofA and Act15 is …KCTKI*GSSGSSGSSGSSQG*DGEDV…, where the italics between the C-terminal sequence of CofA and the N-terminal sequence of Act15 are inserted linker residues.

### Preparation of Proteins

The preparation of cofilin-actin fusion protein was performed as described previously for actin-S1 fusion protein^21^ with some modifications. Briefly, KAx3 wild type *Dictyostelium* cells were transfected with pTIKL cofilin-AR and grown in HL5 medium containing 40 μg/mL G418. The cells were harvested, washed and resuspended in buffer A (20 mM Hepes pH7.4, 0.5 M NaCl, 2 mM MgCl_2_, 1 mM ATP, 7 mM β-mercaptoethanol, 5 mM imidazole pH 7.4 and protease inhibitors). The suspension was mixed with an equal volume of buffer A containing 0.5% Triton X-100, kept for 5 min on ice, and centrifuged at 36,000× g for 30 min at 4 ^o^C. Cofilin-actin in the supernatant was enriched using a Ni^2+^-NTA affinity column (Qiagen, Limburg, Netherlands). The eluate was loaded onto an anti-FLAG M2 monoclonal antibody agarose affinity gel column (Wako, Osaka, Japan), and the column was washed with buffer B (400 mM NaCl, 10 mM Hepes pH 7.4, 1 mM MgCl_2_, 0.5 mM ATP and 7 mM β-mercaptoethanol). Cofilin-actin was eluted with 0.1 mg/ml FLAG peptide dissolved in buffer B and was dialyzed against G-buffer (2 mM Hepes pH 7.4, 0.2 mM CaCl_2_, 0.1 mM ATP and 0.2 mM DTT) containing 10% sucrose. His-tagged *Dictyostelium* cofilin and *Dictyostelium* actin (wild-type actin and G146V mutant actin) were purified as described previously^36^,^37^.

Labeling of cofilin-actin or control actin with AlexaFluor-488 (or −594) succinimidyl ester (Invitrogen, Tokyo, Japan) was carried out as described previously^37^. Labeling of control actin with (*N*-(1-pyrene)iodoacetamide (Invitrogen, Tokyo, Japan) was carried out as described previously^38^.

### Fluorescence Microscopy

The mixture of AlexaFluor-594 labeled control actin (2 μM) and AlexaFluor-488 labeled cofilin-actin fusion protein (1 μM) was polymerized in buffer C (25 mM KCl, 10 mM Pipes pH 6.5, 0.4 mM EGTA, 2 mM MgCl_2_, 0.2 mM ATP and 1 mM DTT) or buffer D (100 mM KCl, 20 mM Hepes pH 7.4, 0.4 mM EGTA, 2 mM MgCl_2_, 0.2 mM ATP and 1 mM DTT) at 22 °C for 30 min. The resultant actin filaments were observed with a fluorescence microscope (BX60, Olympus, Japan) equipped with an EB-CCD camera (C7190, Hamamatsu Photonics, Hamamatsu, Japan) at 25 °C.

### Measurement of Fluorescence

#### Dictyostelium

actin (4 μM) or a mixture of actin (4 μM) and cofilin-actin fusion protein (4 μM) were polymerized in buffer (30 mM KCl, 2 mM Hepes pH 7.4, 0.4 mM EGTA, 2 mM MgCl_2_, 0.2 mM ATP and 1 mM DTT) at 22 °C for 30 min, and then 500 mM Pipes pH 6.5 and 33 μM rhodamine-phalloidin were added at a final concentration of 20 mM and 1 μM, respectively. After 30 min of incubation, fluorescence emission spectra were measured at 22 °C using a spectrofluoro-photometer (RF-5300PC; Shimadzu, Kyoto, Japan) with an excitation wavelength of 549 nm.

Mixtures of 3 μM actin and various concentrations of the fusion protein were polymerized in buffer (100 mM KCl, 2 mM Hepes pH 7.4, 0.4 mM EGTA, 2 mM MgCl_2_, 0.2 mM ATP, 2 mg/ml BSA and 1 mM DTT) at 22 °C for 30 min, and then Pipes pH 6.5  as added (final 20 mM). After 30 min of incubation, resultant actin filaments were diluted 2.8 fold in buffer (10 mM Pipes pH 6.5, 2 mM MgCl_2_, 0.2 mM ATP and 5 mM DTT), and then pyrene fluorescence was monitored at 22 °C using the spectrofluoro-photometer with excitation and emission wavelengths of 365 and 407 nm, respectively.

### Subtilisin Cleavage Assay

Filaments polymerized in buffer (30 mM KCl, 2 mM Hepes pH 7.4, 0.4 mM EGTA, 2 mM MgCl_2_, 0.2 mM ATP and 1 mM DTT) for 30 min at 22 °C were digested by 5 μg/ml or 8.3 μg/ml subtilisin (Sigma) at 25 °C. The reactions were stopped by addition of 1 mM phenylmethylsulfonyl fluoride, and the samples were analyzed by SDS-PAGE. Densitometric analysis was performed with ImageJ version 1.46 software.

### Cosedimentation Assay

*Dictyostelium* actin (3 μM), or a mixture of 3 μM actin and 1.5 μM cofilin-actin fusion protein, were polymerized in buffer (160 mM KCl, 2 mM Hepes pH 7.4, 0.4 mM EGTA, 2 mM MgCl_2_, 0.2 mM ATP and 1 mM DTT) at 22 °C for 30 min, and then Pipes pH 6.5 was added (final 20 mM). After 30 min of incubation, *Dictyostelium* cofilin was added at a final concentration of 3 μM. The mixtures were centrifuged at 250,000× *g* for 10 min at 22 °C, and the supernatants and pellets were subjected to SDS-PAGE. Densitometric analysis was performed as above.

### Electron Microscopy

Cofilin-actin fusion protein and control actin filaments in EM buffer (10 mM potassium phosphate buffer pH 7.4, 25 mM KCl, 2.5 mM MgCl_2_, 0.2 mM ATP, and 0.5 mM DTT) were placed on carbon-coated copper grids, stained with 1% uranyl acetate, and observed in an FEl Tecnai F20 electron microscope. The images were recorded with an ORIUS SC600 slow-scan CCD camera at a magnification of x 50,000, and Gaussian-filtered to reduce noise.

## Additional Information

**How to cite this article**: Umeki, N. *et al*. Cofilin-induced cooperative conformational changes of actin subunits revealed using cofilin-actin fusion protein. *Sci. Rep.* **6**, 20406; doi: 10.1038/srep20406 (2016).

## Supplementary Material

Supplementary Information
