# Supplementary material for: Cofilin-induced cooperative conformational changes of actin subunits revealed using cofilin-actin fusion protein
Source: Sci Rep. 2016 Feb 4;6:20406. doi: 10.1038/srep20406 (PMC4740740; doi:10.1038/srep20406)
Supplement: Supplementary Information [file srep20406-s1.pdf]

Supplementary Information

Cofilin-induced cooperative conformational changes of actin subunits revealed using cofilin-actin fusion protein.

Nobuhisa Umeki[1,3,*], Keiko Hirose[1,2] and Taro Q.P. Uyeda[1,2]

1: Biomedical Research Institute, National Institute of Advanced Industrial Science and Technology, Tsukuba, Ibaraki 305-8562, Japan.
2: Graduate School of Life and Environmental Sciences, University of Tsukuba, Ibaraki 305-8572, Japan.
3: Cellular Informatics Lab., RIKEN, Wako, Saitama 351-0198, Japan.

* Correspondence: Dr. Nobuhisa Umeki
Cellular Informatics Lab., RIKEN, Wako, Saitama 351-0198, Japan.
Phone: +81-48-467-9538;
Fax: +81-48-462-4671;
E-mail: nobuhisa.umeki@riken.jp

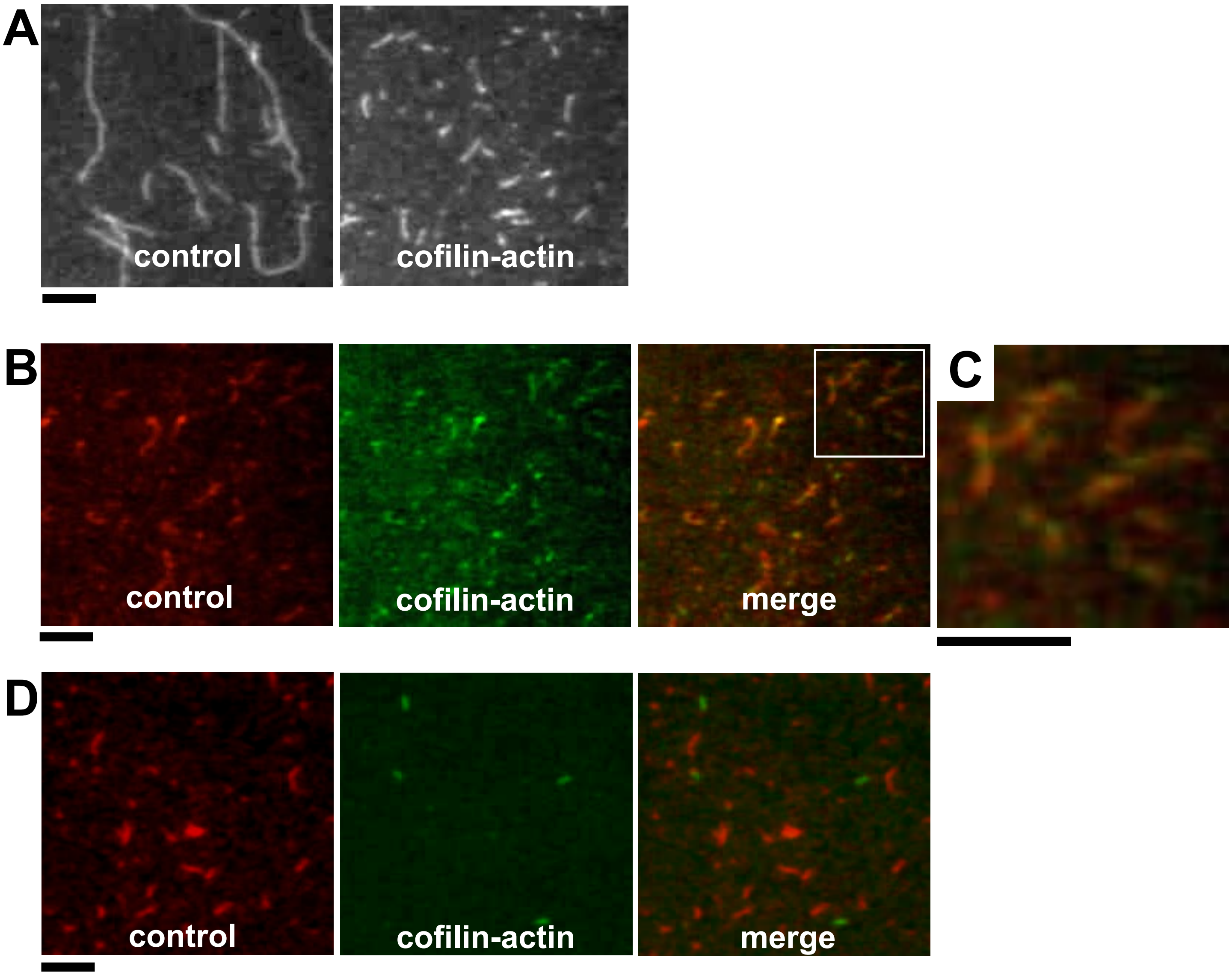

Supplementary Figure S1. Polymerization of the cofilin-actin fusion protein assayed by fluorescence microscopy. (A) Fluorescence micrographs of Alexa Fluor594-labeled control actin and Alexa Fluor488-labeled cofilin-actin fusion protein in buffer (100 mM KCl, 10 mM Pipes pH 6.5, 2 mM $MgCl_2$, 0.4 mM EGTA and 0.2 mM ATP). (B) Copolymerization of Alexa Fluor594-labeled control actin and Alexa Fluor488-labeled cofilin-actin. The two proteins were mixed in G-buffer and then allowed to polymerize at 100 mM KCl and pH 7.4 (in buffer D). (C) is a magnified view of the boxed area in (B). (D) Lack of copolymerization when Alexa Fluor594-labeled control actin and Alexa Fluor488-labeled cofilin-actin were allowed to polymerize at 25 mM KCl and pH 6.5 (in buffer C). Scale bars: 5 μm.

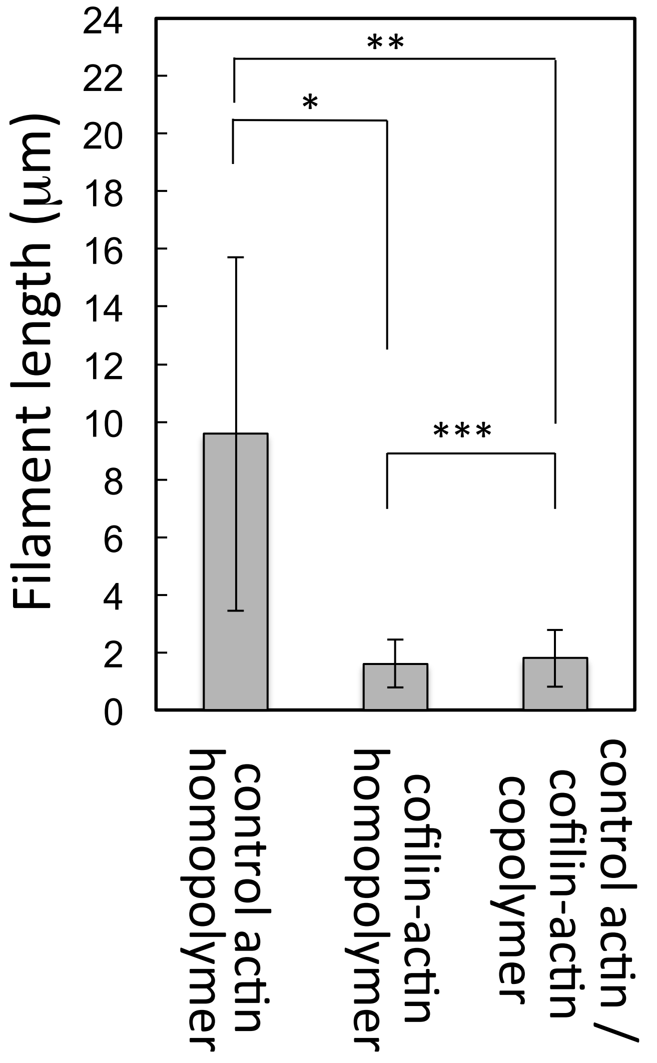

Supplementary Figure S2. Quantitation of filament length. The filament length of control homopolymers, cofilin-actin homopolymers and copolymers were estimated from fluorescent microscopic images shown in Figure 2. Error bars indicate standard deviations (control actin homopolymer, n=172; cofilin-actin homopolymer, n=134; copolymer, n=210). Student's t test: *$P<10^{-37}$, **$P<10^{-36}$, ***$0.05<P<0.10$

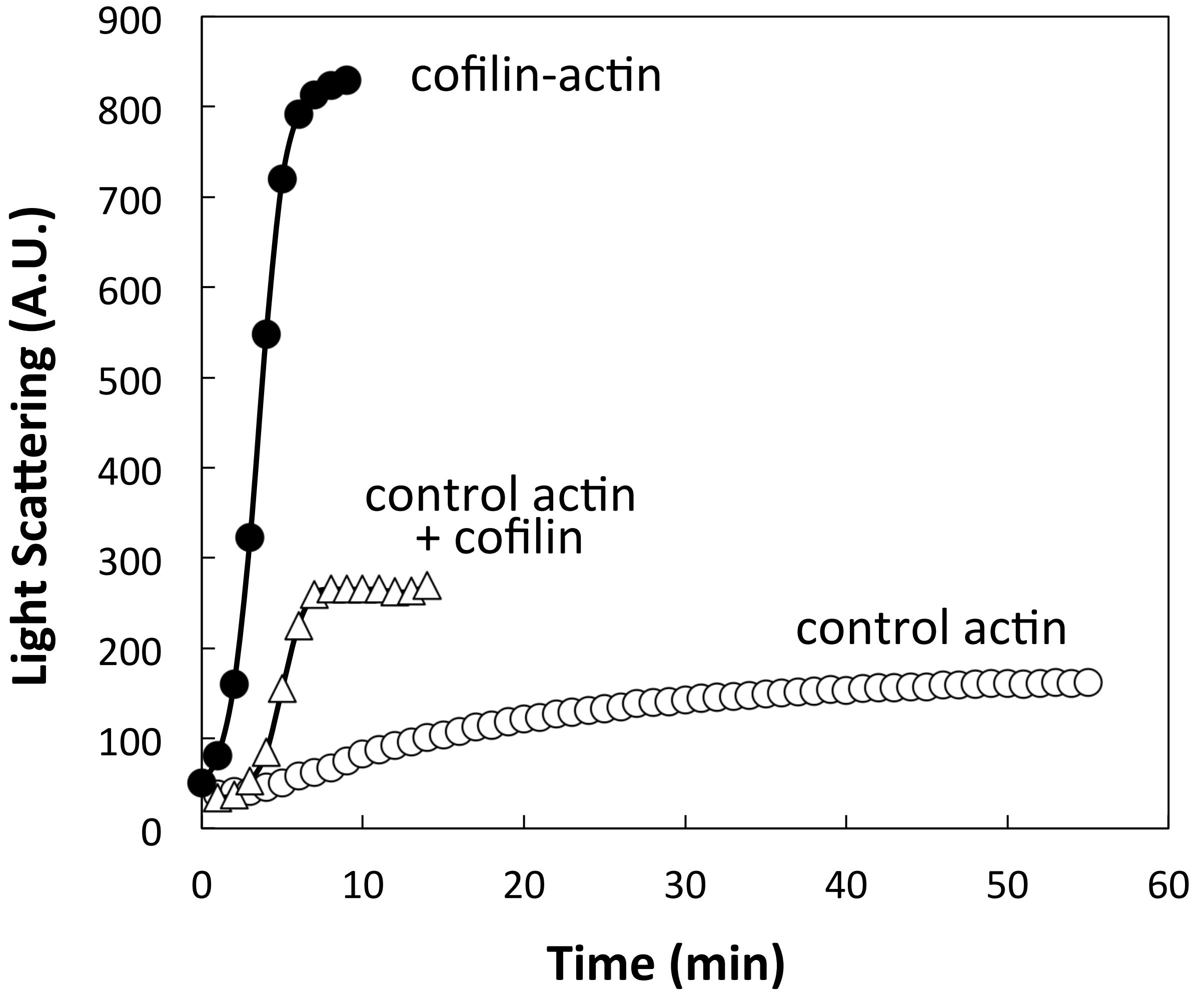

Supplementary Figure S3. Polymerization kinetics of cofilin-actin fusion protein. Cofilin-actin (closed circles), control actin (open circles) and control actin in the presence of cofilin (open triangles) were individually polymerized by the addition of concentrated salts at pH 6.5 and at 22°C. Final reaction mixture contained 1.5 μM actin or cofilin actin, 3 μM cofilin (open triangles only), 50 mM KCl, 15 mM Pipes pH 6.5, 0.5 mM EGTA, 2.5 mM $MgCl_2$, 1 mM ATP and 0.5 mM DTT, and polymerization was monitored by the increase of light scattering at 360 nm. Samples containing cofilin-actin scattered light more intensely than control actin homopolymers, which is presumably in part due to the increased thickness of the filaments.

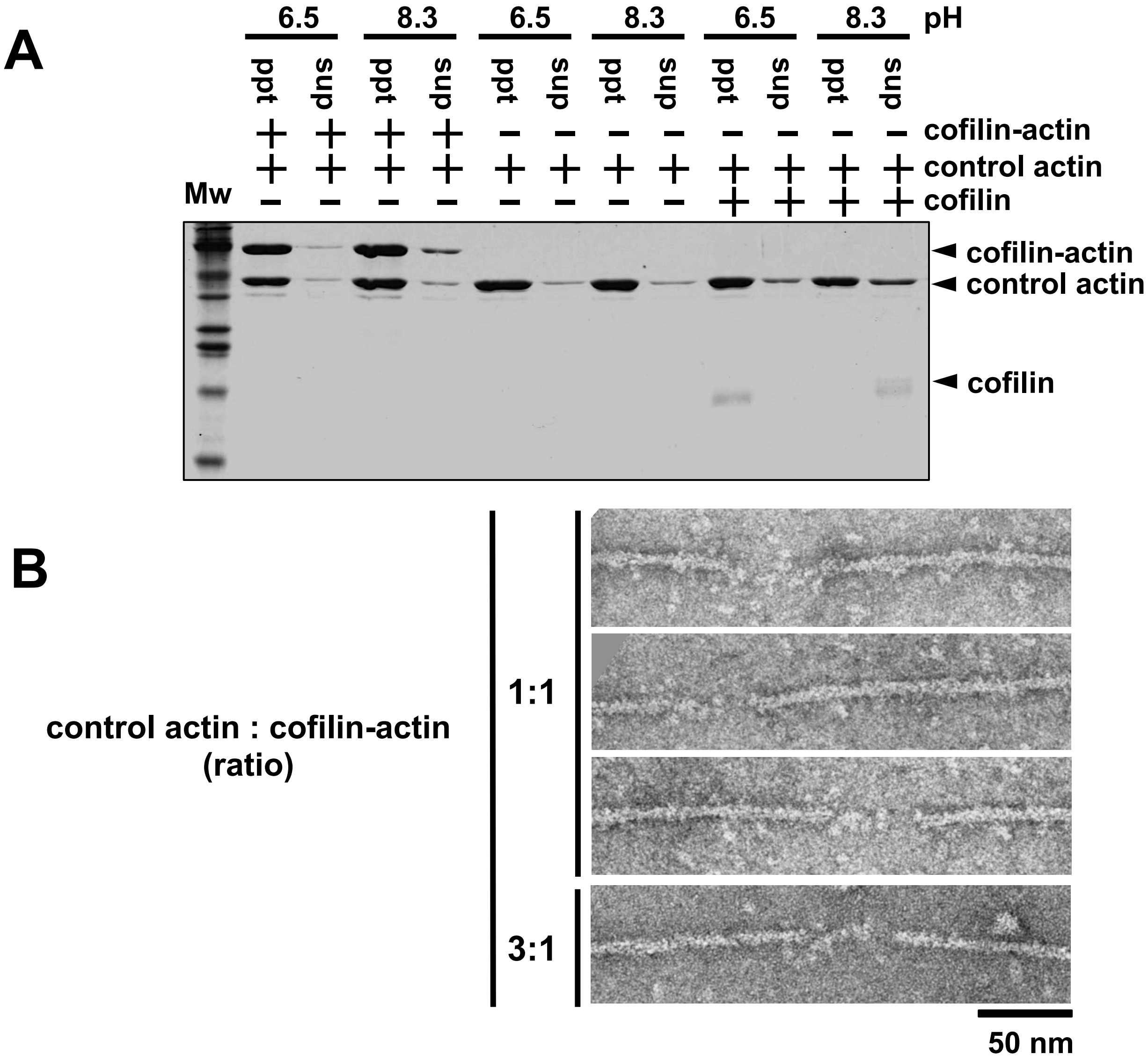

Supplementary Figure S4. Cofilin moiety in the fusion protein has severing and depolymerization activities. (A) Depolymerization of copolymer, control actin homopolymer in the absence of cofilin, and control actin homopolymer in the presence of 4 μM cofilin. *Dictyostelium* actin (10 μM), or a mixture of 10 μM actin and 10 μM cofilin-actin fusion protein, were polymerized in buffer (100 mM KCl, 2 mM Hepes pH 7.4, 0.4 mM EGTA, 2 mM $MgCl_2$, 0.2 mM ATP and 1 mM DTT) at 22°C for 30 min, and then concentrated Pipes pH 6.5 was added (final 10 mM). After 60 min of incubation, an equal volume of 40 mM Pipes buffer (pH6.5) or 40 mM Hepes buffer (pH8.3) was then added and incubated for 2 h at room temperature. Those samples were subjected to ultracentrifugation (250,000 x g for 10 min at 22°C) followed by SDS-PAGE of supernatant and pellet fractions. In the case of copolymers, incubation at pH 8.3 increased the amount of the fusion protein in the sup, but that of control actin was unaffected. This is in contrast to the case of control actin with added cofilin, and is presumably because control actin was able to repolymerize in the absence of cofilin. (B) Electron micrographs of negatively stained copolymers at pH 6.5. Some copolymer filaments showed discontinuities, but the similar gaps were not observed in the control actin homopolymers.

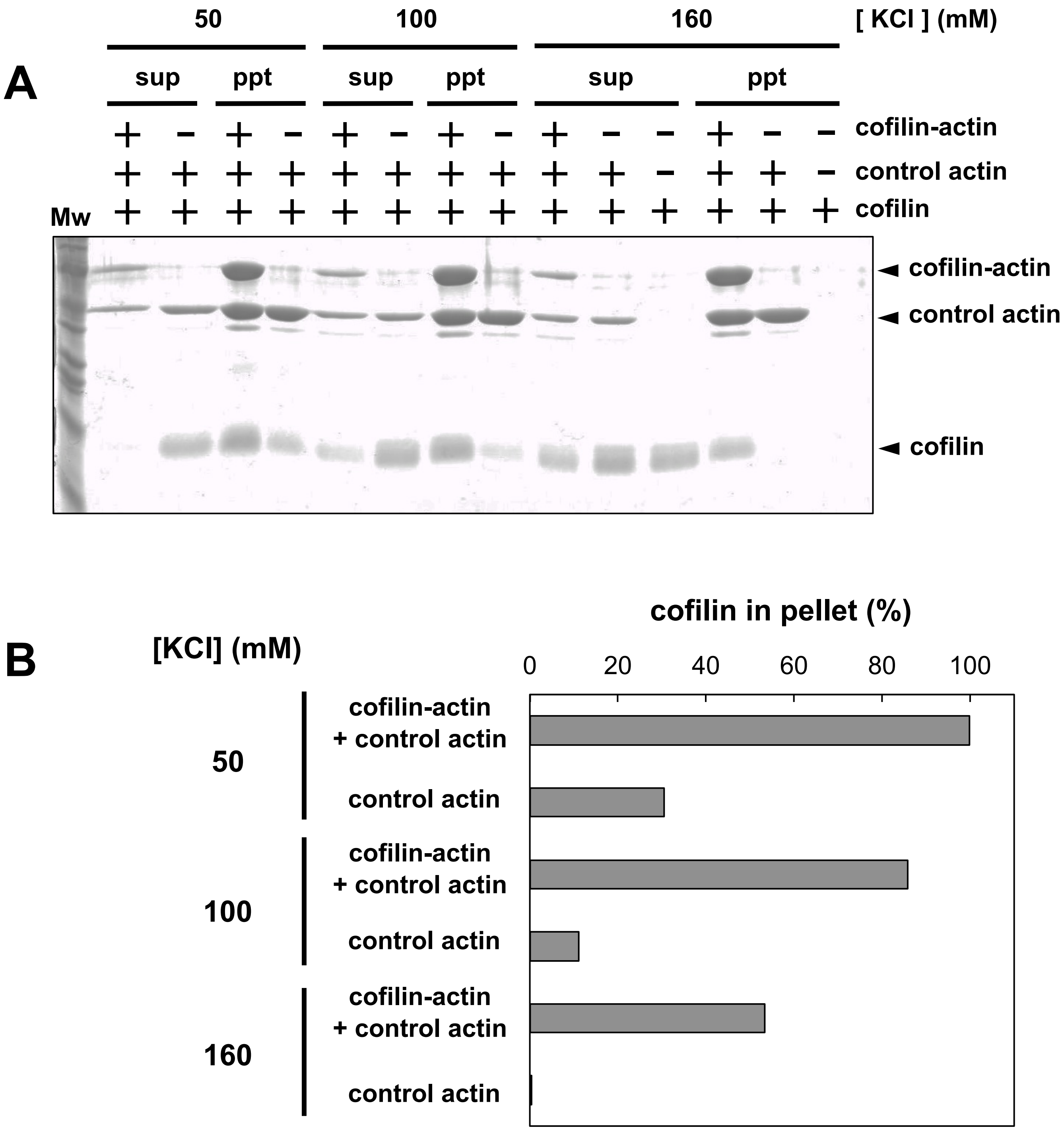

Supplementary Figure S5. Effects of ionic strength on the cooperative binding of cofilin. (A) Cosedimentation assay was performed as described in Methods. (B) Densitometric quantitation. At all three different concentrations of KCl, enhanced binding of cofilin to the copolymers was observed.
